# Identifying Risk Factors Associated With Lower Back Pain in Electronic Medical Record Free Text: Deep Learning Approach Using Clinical Note Annotations

**DOI:** 10.2196/45105

**Published:** 2023-08-09

**Authors:** Aman Jaiswal, Alan Katz, Marcello Nesca, Evangelos Milios

**Affiliations:** 1 Faculty of Computer Science, Dalhousie University, Halifax, NS, Canada; 2 Manitoba Centre for Health Policy, Department of Community Health Sciences, University of Manitoba, Winnipeg, MB, Canada

**Keywords:** machine learning, lower back pain, natural language processing, semantic textual similarity, electronic medical records, risk factors, deep learning

## Abstract

**Background:**

Lower back pain is a common weakening condition that affects a large population. It is a leading cause of disability and lost productivity, and the associated medical costs and lost wages place a substantial burden on individuals and society. Recent advances in artificial intelligence and natural language processing have opened new opportunities for the identification and management of risk factors for lower back pain. In this paper, we propose and train a deep learning model on a data set of clinical notes that have been annotated with relevant risk factors, and we evaluate the model’s performance in identifying risk factors in new clinical notes.

**Objective:**

The primary objective is to develop a novel deep learning approach to detect risk factors for underlying disease in patients presenting with lower back pain in clinical encounter notes. The secondary objective is to propose solutions to potential challenges of using deep learning and natural language processing techniques for identifying risk factors in electronic medical record free text and make practical recommendations for future research in this area.

**Methods:**

We manually annotated clinical notes for the presence of six risk factors for severe underlying disease in patients presenting with lower back pain. Data were highly imbalanced, with only 12% (n=296) of the annotated notes having at least one risk factor. To address imbalanced data, a combination of semantic textual similarity and regular expressions was used to further capture notes for annotation. Further analysis was conducted to study the impact of downsampling, binary formulation of multi-label classification, and unsupervised pretraining on classification performance.

**Results:**

Of 2749 labeled clinical notes, 347 exhibited at least one risk factor, while 2402 exhibited none. The initial analysis shows that downsampling the training set to equalize the ratio of clinical notes with and without risk factors improved the macro–area under the receiver operating characteristic curve (AUROC) by 2%. The Bidirectional Encoder Representations from Transformers (BERT) model improved the macro-AUROC by 15% over the traditional machine learning baseline. In experiment 2, the proposed BERT–convolutional neural network (CNN) model for longer texts improved (4% macro-AUROC) over the BERT baseline, and the multitask models are more stable for minority classes. In experiment 3, domain adaptation of BERTCNN using masked language modeling improved the macro-AUROC by 2%.

**Conclusions:**

Primary care clinical notes are likely to require manipulation to perform meaningful free-text analysis. The application of BERT models for multi-label classification on downsampled annotated clinical notes is useful in detecting risk factors suggesting an indication for imaging for patients with lower back pain.

## Introduction

Lower back pain (LBP) is recognized as a common disability worldwide [1-3]. While there is no agreed-upon definition of LBP, in a systematic review, it was primarily defined through routinely collected electronic health data, which include *International Classification of Diseases, Ninth Revision* (*ICD-9*) and *International Statistical Classification of Diseases, Tenth Revision* (*ICD-10*) codes [4]. One estimate of the burden of LBP is that 13% of adults in the United States live with LBP, while in Canada, among those living with chronic pain, 50.9% identified the location of their pain in the upper or lower back [2,3]. In a systematic review [4], the mean prevalence of LBP among the studies collected ranged between 1.4% and 15.6%.

While the burden of LBP remains high, it is important to understand the indicators for possible serious underlying causes that require imaging, also known as “risk factors” [5]. According to Choosing Wisely Canada, risk factors may include [6]:

- A history of cancer
- Unexplained weight loss
- A recent infection
- Fever
- Loss of bowel or bladder control
- Abnormal reflexes or the loss of muscle power in the legs

Radiological (diagnostic) imaging includes procedures such as x-rays, computed tomography scans, or magnetic resonance imaging scans. Recommendations from clinical practice guidelines state that, unless risk factors are present, radiological imaging is not needed for patients with LBP [5,7]. Moreover, ordering radiological imaging when it is unnecessary puts the patient at risk for radiation exposure and other negative consequences [5,6]. Despite these recommendations, patients with LBP are frequently subjected to unnecessary imaging [8].

The data for this study in clinical practice uses electronic medical records (EMRs). The widespread use of this IT has introduced the feasibility of analyzing large numbers of clinical notes without having to manually access paper charts and perform the analyses using automated approaches such as natural language processing (NLP) [9]. The Canadian Primary Care Sentinel Surveillance Network [10] routinely extracts clinical information such as clinical encounter notes, note type, and the date of the notes from primary care clinical practices with the permission of the providers. Applying NLP methods to EMR data makes it possible to detect LBP risk factors and understand the use of imaging in this common clinical presentation.

Since the introduction of transformers in 2019 [11], which are large language models that can be fine-tuned for specific tasks, deep language models have achieved a significant milestone in natural language understanding. The transfer learning paradigm of unsupervised pretraining and fine-tuning [12] using Bidirectional Encoder Representations from Transformers (BERT) has reduced the requirement for large labeled data sets to achieve state-of-the-art analytic performance. Previous research [13] has explored the use of topic models and deep neural networks to automatically distinguish acute LBP episodes using free-text clinical notes.

## Methods

The following steps were undertaken to achieve our goal: preparation of EMR data, EMR annotation process, addressing imbalanced data, and application of the proposed model.

### Preparation of EMR Data

We accessed a random sample of deidentified EMR data, and using the regular expressions created in SAS (SAS Institute), we identified a cohort of patients with any indication of LBP. Notes were further filtered by note type to only include provider-generated clinical notes. The data were then split randomly into three files. Ethics approval for the study was provided by the University of Manitoba Health Research Ethics Board and the Health Information Privacy Committee.

### EMR Annotation Process

Six medical students reviewed the EMR notes to identify the six LBP risk factors in accordance to Choosing Wisely Canada. They worked in teams of two to validate the application of the inclusion and exclusion criteria, each note being annotated by two students. The inclusion criteria listed in Textbox 1 were the presence of specific clinical notes suggestive of at least one of the six risk factors indicating the need for imaging. The exclusion criteria were the presence of clinical conditions that could lead to symptoms that may be confused with any of the underlying conditions represented by the six risk factors and clinical notes that do not represent relevant visits.

Textbox 1. **Inclusion and exclusion criteria for risk factors.**

**Inclusion criteria**

- Lower extremities for loss of muscle function
- Positive straight leg test
- Nerve impingement
- Sciatica, but need to confirm radiculopathy
- Incontinence related to a nerve issue
- If back pain has improved
- Follow-up discussions of imaging results
- Saddle anesthesia
- Notes that do not specify upper vs lower back pain

**Exclusion criteria**

- HIV is not a relevant infection (regardless of viral load and strain/location)
- Urinary symptoms other than incontinence are neither risk factors nor symptoms of relevant infection
- Shingles as an infection if it is a lumbar dermatome
- Nocturnal enuresis
- Degenerative diseases or osteoarthritis with an indication of back pain
- Copy/pasted imaging results onto the electronic medical record note
- Notes that mention previous or resolved back pain
- Well child/adolescent visit

An experienced clinician (AK) arbitrated any disagreements between student annotators. This supported the inclusion of correctly labeled records in the classification model. For the annotation process, we used Microsoft Forms (Microsoft Corporation), which enabled us to collect the relevant data in a systematic and organized manner. Specifically, the output from Microsoft Forms was linked to a secure CSV file containing the clinical notes, using a unique identifier to facilitate data merging and subsequent analysis.

### Addressing Imbalanced Data

Our data collection process consisted of two rounds. In the first round, we established the initial distribution of risk factors. Analysis of this round revealed an imbalanced distribution of labels, a well-known factor that can impact the performance of deep learning methods [14,15]. Specifically, we observed an imbalance in both the infrequent occurrence of individual risk factors and the high frequency of the “null class,” which denotes the absence of risk factors.

To address this imbalance, we adopted a 2-pronged approach. First, we collected additional clinical notes specifically targeting minority risk factors. Second, we downsampled the majority of notes with “null class.” Notably, the initial data set lacked any clinical notes for unexplained weight loss. Table 1 depicts the distribution of risk factors after the first labeling round, revealing that only 12% (n=296) of the 2487 annotated notes exhibited any risk factors.

**Table 1.** Risk factor distribution after the first labeling round. Zero notes exhibit the unexplained weight loss risk factor.

Risk factors	Annotations (round 1), n
Cancer	26
Weight^a^	0
Fever	8
Infection	8
Bowel	9
Abreflex	233

a Zero notes exhibit the unexplained weight loss risk factor.

### Acquiring More Notes to Annotate

Prior studies have explored methods for addressing the challenge of obtaining sufficient data for training [16]. To acquire clinical notes for labeling that are more likely to exhibit a minority risk factor, we used unsupervised semantic textual similarity (STS). It is a ranking task where given a text query and a list of clinical notes, the STS model ranks the clinical notes that are semantically like the query. We trained two unsupervised STS models, Transformers and Sequential Denoising Auto-Encoder (TSDAE) [17] and Simple Contrastive Learning of Sentence Embeddings (SimCSE) [18], implemented using the SentenceTransformer Python library [19]. To rank the unlabeled clinical notes (ie, 55,000 notes with any LBP indication), we formed the queries using rationales, collected as part of the first labeling round. Here, we refer to “rationale” as an extracted snippet or text from the clinical note the annotators highlighted as evidence for a risk factor.

**Figure 1.**
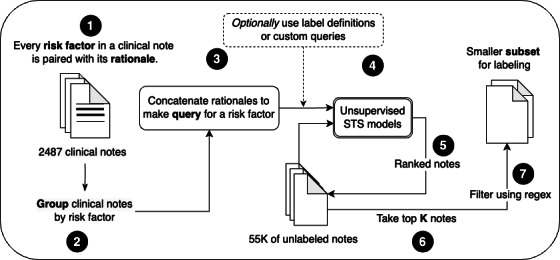
Semantic textual similarity sampling process, followed for the second labeling round. STS: semantic textual similarity.

Figure 1 illustrates the STS sampling process with numbered steps. First, we group the clinical notes based on the exhibited risk factors. We then concatenate the rationales for each group of clinical notes to form queries and rank the unlabeled clinical notes using the unsupervised STS models. If the rationales were unavailable from the first labeling round (eg, “weight loss”), we used risk factor definition or custom text as the query. We selected the top K notes from the ranked clinical notes, where “K” is set within the 10-50 range. We further filtered noisy outputs using phrases such as “has fever,” “has back pain,” and “lost weight.” Finally, we iterated the process for each risk factor and provided the selected notes for the second labeling round.

This approach helped maximize annotations for clinical notes that exhibited risk factors. Table 2 depicts the complete distribution of risk factors after both rounds of labeling. Of the 262 annotated clinical notes in the second round, 19.5% (n=51) of the clinical notes exhibited risk factors, in contrast to 12% (n=296) in the first round.

**Table 2.** Risk factor distribution after both rounds of labeling.^a^

Risk factors	Annotations (round 1 + 2), n
Cancer	53
Weight	32
Fever	17
Infection	9
Bowel	9
Abreflex	236

a This includes 2487 notes from the first round and 262 notes from the second round. In the second labeling round, we collected 32 clinical notes for the unexplained weight loss risk factor.

### Treating Class Imbalance With Downsampling

Following the second round of labeling, a significant class imbalance was observed in the resulting distribution of labels. Specifically, out of the total 2749 annotated clinical notes, only 347 were labeled as having one or more risk factors, while the remaining 2402 notes were labeled with no risk factor. To mitigate this issue, two common approaches are oversampling the minority class or downsampling the majority class. In a multi-label data set, each instance can be assigned to one or more classes. For instance, in the case of clinical notes, they may have one or more risk factors, making it challenging to oversample the minority class. This is because generating synthetic instances requires randomly selecting a minority clinical note that may have a combination of labels rather than a single label. However, this approach may bias the model toward the minority class and lead to overfitting. Consequently, we opted for downsampling the majority class to balance the class distribution and prevent the model from being biased toward the majority class.

Specifically, a subset of the clinical notes with “no risk factors” was randomly selected to match the number of clinical notes with “any risk factor.” This approach aimed to balance the class distribution and enable the model to learn from both positive and negative examples. To assess the effectiveness of the downsampling strategy, we conducted a comparative analysis of the model’s performance with and without downsampling.

### Application of Proposed Model

**Figure 2.**
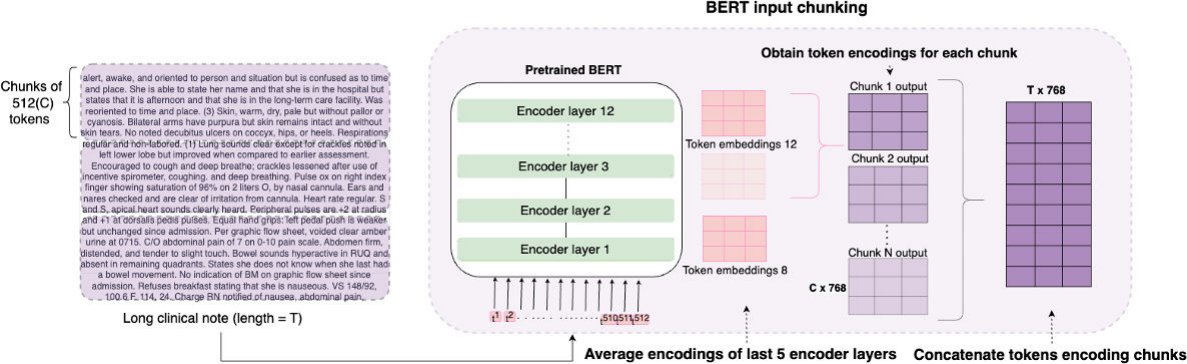
BERT input chunking: a clinical note is first separated into chunks of 512 tokens. Each chunk is then independently processed by the BERT-based back-end model. The chunk embedding is obtained by averaging the token embeddings from the last five layers of BERT. Finally, all the chunk embeddings are concatenated and processed using convolution layers, as defined by Kim [22]. Note: The sample clinical note does not belong to the real data set. BERT: Bidirectional Encoder Representations from Transformers.

Transformer-based BERT [11] models can be fine-tuned for detecting risk factors in clinical notes using a small labeled data set. The requirement for large labeled data sets is eased with models that are pretrained on large clinical text. In this work, we used BlueBERT [20] as our back-end model that is pretrained on PubMed abstracts and clinical notes from the Medical Information Mart for Intensive Care (MIMIC-III) data set [21]. However, BERT models are limited to a maximum input length of 512 tokens. The length of clinical notes in our data set ranges from 7 to 1400 tokens with 8% (n=221) of the notes having more than 512 tokens. To overcome this limitation, we propose a novel architecture called BERT–convolutional neural network (CNN) that chunks the inputs and processes them using convolution layers. The proposed chunking method is illustrated in Figure 2.

### Experimental Setup

The study used a repeated 2-fold cross-validation approach with two repetitions to improve the estimated performance of the machine learning models. As the data set was multi-label, we adopted the iterative stratification method [23,24] provided by the scikit-multilearn library [25] to generate stratified splits for the folds. This ensured that every split had a similar distribution of risk factors. The 2-fold cross-validation was repeated twice, resulting in a total of four runs. Wherever applicable, we implemented the downsampling technique (as described earlier) on the training set. Our results are reported in terms of the area under the receiver operating characteristic curve (AUROC) of individual risk factors and their macroaverage across the folds. Table 3 reports the frequency of positive risk factors in each split of the folds.

**Table 3.** Frequency of positive risk factors in train-test splits. We report the approximate counts of each risk factor across folds. Note: the counts do not include the clinical notes with no risk factors, which are approximately 1198 and 1195 for the train and test split, respectively.

Positive risk factors	Train split (n=1374 notes), n	Test split (n=1375 notes), n
Cancer	26	27
Weight	16	16
Fever	8	9
Infection	4	5
Bowel	4	5
Abreflex	118	118

### Ethics Approval

The study received ethics approval from the Health Research Ethics Board of the University of Manitoba (study number HS20263; review number H2016:408).

## Results

### Overview

In this section, we report the analysis of the data collection and classification performance of the transformer-based models with different configurations, including traditional machine learning and BERT-based baselines. The transformer-based models were trained for 10 epochs each, with a learning rate ranging from 5e-05 to 6e-5. Unless specified otherwise, all the BERT-based models use BlueBERT [20] as the back end.

### Data Collection Analysis

Each annotation was added to the clinical note level independently. These notes are associated with patient- and site-level information, allowing for further analysis based on the patient and site as the unit of analysis. Table 4 presents an analysis of the LBP characteristics reported in the collected data, using notes, patient, and site ID as the units of analysis. This enables a multilevel analysis of the reported characteristics, providing a detailed understanding of their distribution across various units of analysis.

**Table 4.** Lower back pain characteristics gathered from collected data, with notes, patient, and site ID each serving as the units of analysis.

Unit of analysis		Values, n (%)	
**Notes (N=2749)**			
	History of cancer	53 (1.9)	
	Signs of fever	17 (0.6)	
	Unexplained weight loss	32 (1.2)	
	Recent infection	9 (0.3)	
	Loss of bowel or bladder control	9 (0.3)	
	Abnormal reflexes	236 (8.6)	
**Patients (N=1943)**			
	History of cancer	40 (2.1)	
	Signs of fever	17 (0.9)	
	Unexplained weight loss	32 (1.6)	
	Recent infection	9 (0.5)	
	Loss of bowel or bladder control	8 (0.4)	
	Abnormal reflexes	201 (10.3)	
**Site ID (N=22)**			
	History of cancer	12 (55)	
	Signs of fever	11 (50)	
	Unexplained weight loss	12 (55)	
	Recent infection	5 (23)	
	Loss of bowel or bladder control	7 (32)	
	Abnormal reflexes	13 (59)	

A total of 2749 clinical notes were annotated to collect information on risk factors for LBP. The most reported risk factor was “abnormal reflexes,” with 236 annotations, followed by “history of cancer” with 53 annotations. Out of the 1943 patients covered by the annotation process, only 40 were labeled with a “history of cancer,” accounting for 2.1% (n=40) of the total patients. More than 10% of patients were reported with “abnormal reflexes,” while “recent infection” and “loss of bowel control” were reported in only 9 and 8 patients, respectively.

The analysis of clinical sites associated with the clinical notes revealed that 12 of 22 sites reported at least two risk factors, with “recent infection” and “loss of bowel or bladder control” being the least commonly reported risk factors, mentioned in only 5 and 7 clinical sites, respectively. These findings indicate that “abnormal reflexes” is the most reported characteristic of LBP across all units of analysis, with “history of cancer,” “unexplained weight loss,” and “signs of fever” being reported less frequently. The frequency of “loss of bowel or bladder control” and “recent infection” was relatively low across all units of analysis, indicating that these characteristics may not be as common as others in cases of LBP. The distribution of these characteristics varies across different units of analysis, which highlights the importance of examining LBP characteristics at multiple levels.

### Performance With and Without Downsampling

**Figure 3.**
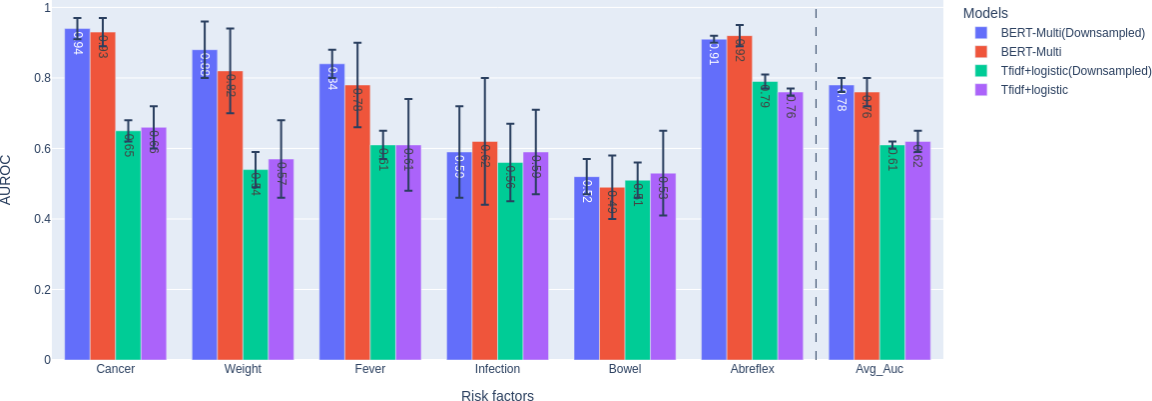
Comparison of BERT-Multitask models trained on complete and downsampled data. A tf-idf + logistic regression model trained with a multi-output classifier is included as a baseline. The AUROC for each risk factor and their macroaverage are reported, with the SDs reflected in the error bars. AUROC: area under the receiver operating characteristic curve; BERT: Bidirectional Encoder Representations from Transformers; tf-idf: term frequency–inverse document frequency.

In our initial analysis, we compared the impact of downsampling the training set, as described earlier, on the average and label-wise performance of the models. Figure 3 displays the results of this comparison. We also included a tf-idf (term frequency–inverse document frequency) + logistic regression model trained with a multi-output classifier [26] as a baseline, which was the best-performing baseline (among 7 candidates, including k-nearest neighbors, naive Bayes, random forest, and models from the scikit-multilearn Library [25]). On average, the BERT models performed 15% better than the baseline. Downsampling the training set improved performance by 2% for BERT-Multi models and reduced the SD as reflected by the error bars for minority labels (eg, “bowel” and “fever”). Downsampling of the majority class (ie, “No Risk factor notes”) also helped stabilize the performance of the models, as indicated by the smaller error bars. We used the downsampled training set for further analysis.

### Performance With BERTCNN and Independent Binary Classifiers

**Figure 4.**
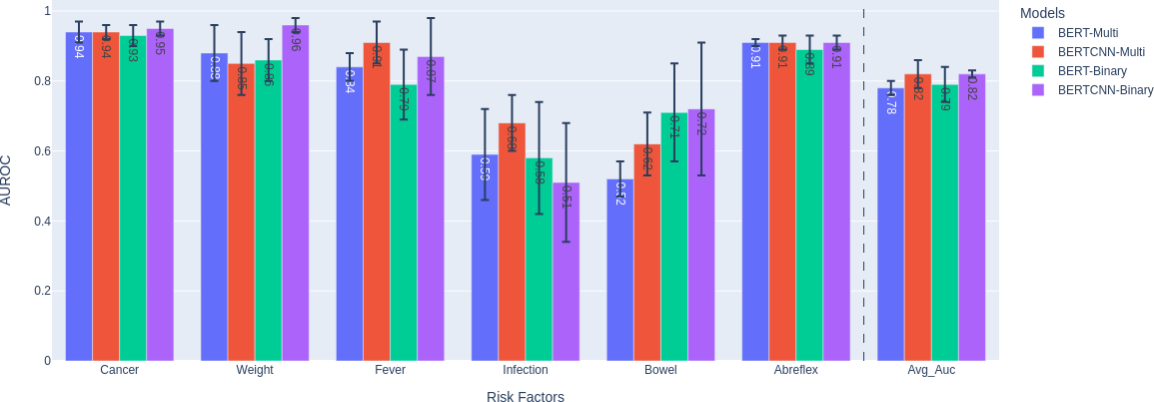
BERT-Multi, BERT-Binary, BERTCNN-Multi, and BERTCNN-Binary trained on the downsampled training data. The AUROC for each risk factor and their macroaverage are reported, with the SDs reflected in the error bars. AUROC: area under the receiver operating characteristic curve; BERT: Bidirectional Encoder Representations from Transformers; BERTCNN: Bidirectional Encoder Representations from Transformers–convolutional neural network.

Using the downsampled training set for all the models, we compared the performance of four different models chosen by architecture (BERT, BERTCNN) and task formulation (multitask learning, binary classification). Figure 4 shows the results. The comparison of BERT and BERTCNN highlights the importance of not truncating longer inputs. The comparison of the proposed model (BERTCNN) with their binary variants helps in understanding the trade-off between parameter efficiency and performance. The average AUROC of all the models are comparable, with BERTCNN-Multi performing 4% better than BERT-Multi. The multitask BERT and BERTCNN models match the performance of their binary alternative with six times fewer parameters. When sufficient positive samples are present for a risk factor (eg, abreflex), all the models perform comparably with a low SD. When the samples are insufficient (eg, “infection” and “bowel”), the binary models have high SD (indicated by the error bars), as few-samples BERT fine-tuning is known to be unstable [27]. In such cases, the multitask models generally produce more stable results, with the BERTCNN-Multi performing 9% better than BERT-Multi. In general, the BERTCNN model can benefit from the extra context found in the complete clinical note to improve prediction performance.

### Performance With Domain Adaptation Using Unsupervised Training

**Figure 5.**
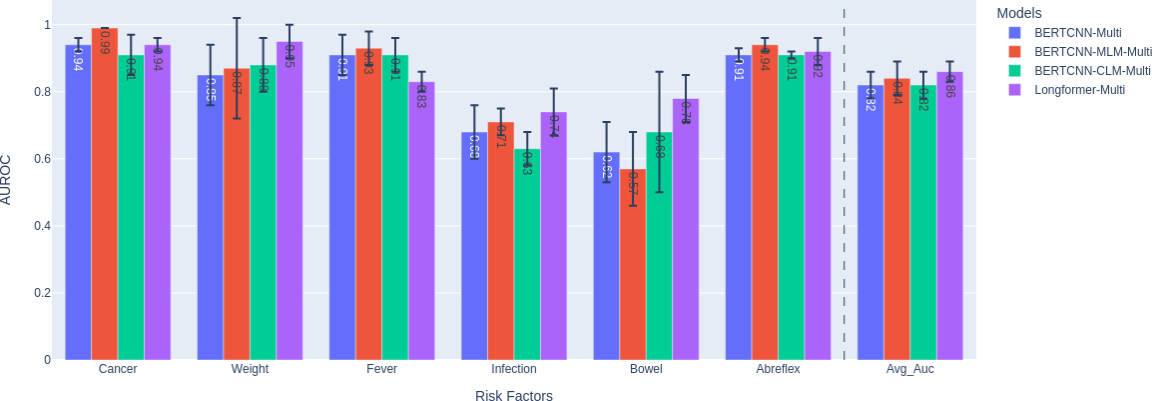
Effect of domain adaptation using MLM, CLM, and comparison with the clinical-longformer model. The AUROC for each risk factor and their macroaverage are reported, with the SDs reflected in the error bars. AUROC: area under the receiver operating characteristic curve; BERTCNN: Bidirectional Encoder Representations from Transformers–convolutional neural network; CLM: causal language modeling; MLM: masked language modeling.

The best-performing model can further benefit from pretraining [28] the underlying transformer model using the clinical notes. In this analysis, we investigate the effect of domain adaptation using pretraining on classification performance. We used BERTCNN and further pretrained the back-end model (BlueBERT [20]) with the complete corpus of relevant clinical notes (N=57,000) for 3 epochs. Two choices for pretraining the BERT architecture were considered: masked language modeling (MLM; BERTCNN-MLM-Multi) [12] and causal language modeling (CLM; BERTCNN-CLM-Multi) [29]. In addition, we also report results of the recent transformers-based model for long text in the clinical domain, called clinical-longformer [30,31], which was pretrained on clinical notes from the MIMIC-III data set [21]. Our results, shown in Figure 5, indicate that the MLM method performed 2% better than no domain adaptation and improved the performance for “cancer” by 5%. The longformer model further improves performance over MLM by 2%. It is worth noting that while the performance improvement of domain adaptation using MLM [32] is not significant, it is comparable to that of the already pretrained BlueBERT [20] and clinical-longformer [30,31], which were pretrained on a much larger corpus of over 2 million notes.

## Discussion

The analysis of electronic clinical notes using machine learning techniques provides the opportunity to explore and evaluate clinical care, previously not possible when clinical experts had to read each clinical record. NLP of clinical records is still a relatively new research endeavor that is rapidly evolving. This study encountered and addressed several challenges that are likely to be common in the analysis of clinical notes. For example, the initially collected data were imbalanced, with most clinical notes having no risk factors for cancer, suggesting the need for further investigation of LBP. By sampling additional clinical notes from the unlabeled pool using unsupervised semantic matching techniques for a limited second round of labeling, we captured 7.5% more clinical notes with at least one risk factor. Strategic resampling can decrease bias in multi-label data sets, which substantially helps in classification performance. The analysis comparing multitask learning and binary classification suggests we can match the performance of independent binary classifiers and produce more stable results while using a fraction of the learned parameters required for binary classifiers. This study demonstrates the value of domain adaptation as an additional technique to improve the classification results of transformer-based models and improve clinical free-text classification using unsupervised methods.

A strength of this study is the comparison of different models and approaches using a random sample of real clinical notes. We compared the BERT-based model, which does not truncate longer clinical notes and uses the complete context to make predictions, to the more commonly used truncated note model. The extensive empirical analysis on the impact of different modeling choices, including comparisons of multitask and single-task learning, resampling of data, and domain adaptation using unsupervised methods for the detection of LBP risk factors in clinical notes, provides guidance for future analysis of clinical text data.

While the low number of samples for certain risk factors in the test set is a limitation, this was addressed in reporting the AUROC for each individual risk factor, including their macroaverage for each model, and using the repeated k-fold cross-validation approach for better estimation of performance.

Future research will involve linking the outcomes of imaging studies to the identification of risk factors in this data set. It is anticipated that patients without risk factors would have normal imaging, while those with risk factors should be more likely to have abnormal imaging suggestive of disease requiring further treatment. Those analyses will need to address the imbalance in the data, as a minority of patients have undergone imaging.

Deep learning models, specifically BERT-based models, are suitable for capturing and detecting risk factors for LBP in clinical notes. Semantic matching techniques are effective during data collection in providing minority samples for labeling and improving data set distribution. The proposed method BERTCNN can be successfully applied for clinical notes that may be longer than the input limit of BERT-based models. Detecting risk factors in clinical notes is better formulated as multitask learning, which is more efficient and provides stable results. Furthermore, transformer-based models are successfully adopted for clinical text using transfer learning and MLM.

## Abbreviations

AUROC: area under the receiver operating characteristic curve
BERT: Bidirectional Encoder Representations from Transformers
BERTCNN: Bidirectional Encoder Representations from Transformers–convolutional neural network
CLM: causal language modeling
EMR: electronic medical record
*ICD-10*: *International Statistical Classification of Diseases, Tenth Revision*
*ICD-9*: *International Classification of Diseases, Ninth Revision*
LBP: lower back pain
MIMIC-III: Medical Information Mart for Intensive Care
MLM: masked language modeling
NLP: natural language processing
SimCSE: Simple Contrastive Learning of Sentence Embeddings
STS: semantic textual similarity
tf-idf: term frequency–inverse document frequency
TSDAE: Transformers and Sequential Denoising Auto-Encoder
